# Commentary: Ultrasound-Guided Triamcinolone Acetonide Hydrodissection for Carpal Tunnel Syndrome: A Randomized Controlled Trial

**DOI:** 10.3389/fmed.2021.833862

**Published:** 2022-01-13

**Authors:** King Hei Stanley Lam, Wai Wah Lai, Ho Yin Ngai, Wing Keung Ricky Wu, Yung-Tsan Wu

**Affiliations:** ^1^The Hong Kong Institute of Musculoskeletal Medicine, Kwoloon, Hong Kong SAR, China; ^2^Department of Family Medicine, The Chinese University of Hong Kong, Shatin, Hong Kong SAR, China; ^3^Department of Family Medicine, The University of Hong Kong, Pokfulam, Hong Kong SAR, China; ^4^Taiwan Association of Prolotherapy and Regenerative Medicine, Taichung, Taiwan; ^5^Center for Regional Anesthesia and Pain Medicine, Wan Fang Hospital, Taipei Medical University, Taipei, Taiwan; ^6^Department of Physical Medicine and Rehabilitation, School of Medicine, National Defense Medical Center, Tri-Service General Hospital, Taipei, Taiwan; ^7^Integrated Pain Management Center, Tri-Service General Hospital, School of Medicine, National Defense Medical Center, Taipei, Taiwan; ^8^Department of Research and Development, School of Medicine, National Defense Medical Center, Tri-Service General Hospital, Taipei, Taiwan

**Keywords:** carpal tunnel syndrome, ultrasound-guided hydrodissection, corticosteroid, nerve entrapment, median nerve (MN)

## Introduction

We read with great interest the recently published paper entitled, “*Ultrasound-Guided Triamcinolone Acetonide Hydrodissection for Carpal Tunnel Syndrome: A Randomized Controlled Trial*” by Wang et al. (1). We appreciate that the authors have performed another randomized control trial on this crucial and frequently encountered median nerve(MN) entrapment in the carpal tunnel syndrome(CTS). It is important that the authors evaluate whether corticosteroid hydrodissection using a larger injectate(s) volume provides additional clinical improvement.

## Commentary

**Figure 1:**
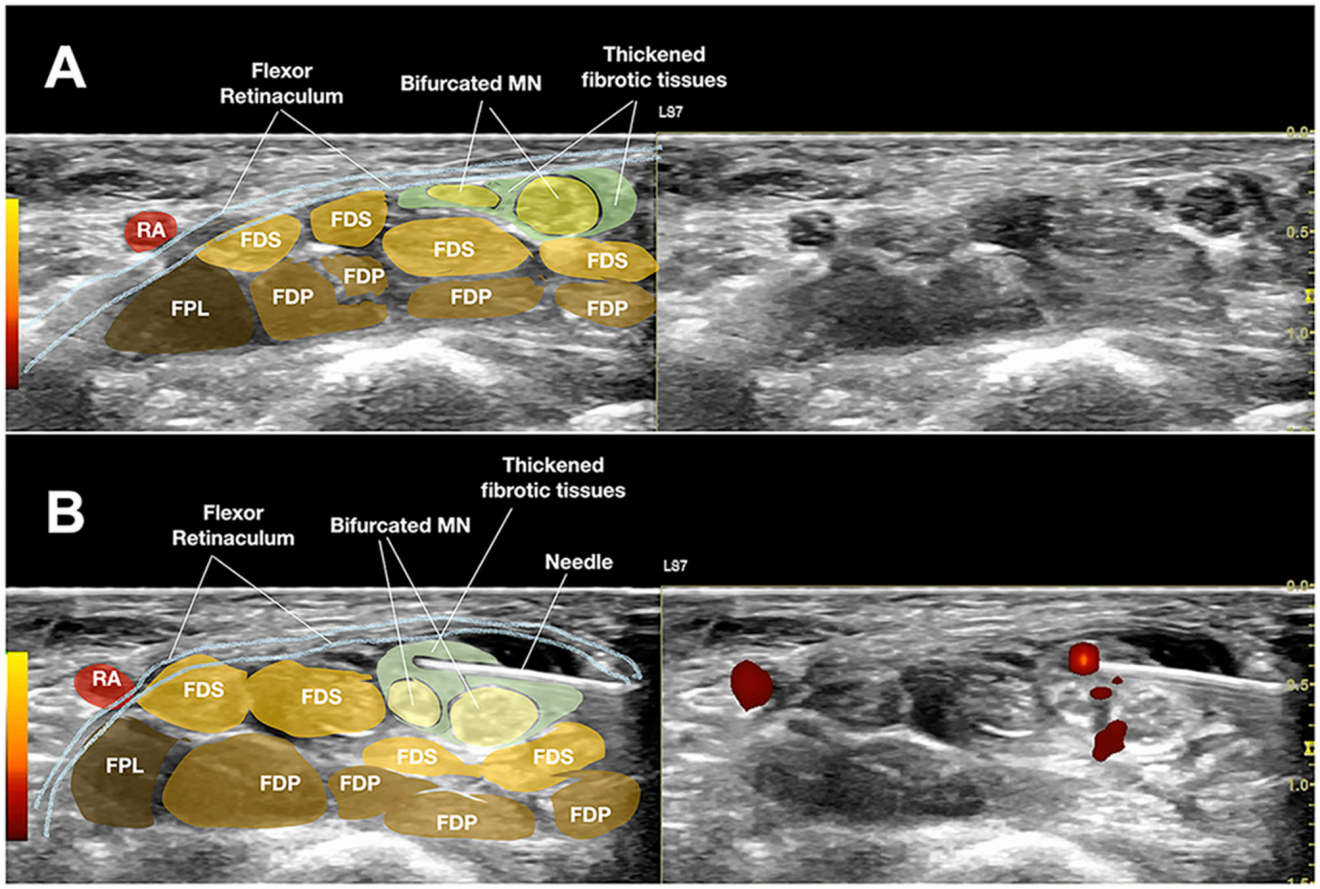
Figure showing the median nerve before and after successful hydrodissection. Before hydrodissection, the median nerve is already bifurcated in the carpal tunnel, and the whole nerve appears elliptical **(A)**. After hydrodissection, both branches of the median nerve have been completely released from the surrounding fibrous/scar tissues, both above and below, ulnar and radial to the nerve branches. Additionally, both median nerve branches looked oval/rounded and surrounded by a halo of fluid as the endpoint **(B)**. FDS, flexor digitorum superficialis tendon; FDP, flexor digitorum profundus tendon; FPL, flexor pollicis longus tendon; MN, median nerve; RA, superficial palmar branch of radial artery.

When reviewing the methods used in this study, we found a factor that might have contributed to the study having no difference in the hydrodissection group relative to the steroid-only perineural injection group. When the authors described the hydrodissection method, they mentioned injecting half of the fluid above the MN and half below the MN, with more fluid accumulated over the ulnar side of the nerve. The authors did not describe the final shape of the nerve after hydrodissection. However, Figure 1B of Wang et al's manuscript noticed that the MN after hydrodissection was still elliptical, which is contrary to our experience and many other researchers' experiences of performing ultrasound-guided hydrodissections (2). All the fibrotic and entrapping scarring tissues outside the epineurium need to be separated and the vasa- and nervi-nervorum must be freed (2, 3). The halo should be equally located on the radial and ulnar sides of the MN, and the final MN should appear rounded/oval instead of elliptical (2, 3) (Figure 1). Moreover, when looking at Figure 1B of Wang's et al. manuscript carefully, the radial side of the nerve still appears to have some hyperechoic fibrotic tissue over the most radial corner of the MN, which might not be the normal mesoneurium and should have been hydrodissected away from the epineurium. This tethering soft tissues may be why the MN was entrapped and compressed between the flexor pollicis longus and other digitorum tendons (4). Moreover, if the author could use the same needle entry point, after hydrodissecting the most entrapped part of the MN, and then pivoted the needle and transducer to a more proximal and thence a more distal part of the MN inside the carpal tunnel and repeated the hydrodissection processes, a longer length of the entrapped MN could have been hydrodissected more completely (2, 3), which could have further enhanced the efficacy in the hydrodissection group. An incompletely hydrodissected nerve may suggest why there was no additional benefit to using hydrodissection.

Another possible systematic error in this study was in the triamcinolone concentration. In the “Methods” section of the abstract, it was stated that “Subjects were randomly assigned to either ultrasound-guided hydrodissection with a mixture of 1mL of triamcinolone acetonide (10 mg/mL), 1 mL of 2% lidocaine, and 8 mL normal saline or ultrasound-guided perineural injection with 1 mL of triamcinolone acetonide (40 mg/mL) and 1 mL of 2% lidocaine.” That is, the first group received 1 mL of 10 mg/mL triamcinolone acetonide and the second group received 1 mL of 40 mg/mL drug. However, the “Methods” section of the text stated, “The injection [group 1, 1 mL 2% lidocaine hydrochloride + 1 mL triamcinolone acetonide (10 mg/mL) + 8 mL normal saline; group 2, 1 mL 2% lidocaine hydrochloride + 1 mL triamcinolone acetonide (10 mg/mL)].” Could the authors kindly confirm the steroid dosage in each group? We believe this could be a very significant confounding variable.

## Discussion

Even with the same concentration of 10 mg/mL triamcinolone added as the injectate of the two patients groups, the final concentration of the triamcinolone reaching the nerve or remaining in the carpal tunnel may be significantly different. First, the final concentration of triamcinolone in group 1 was 1 mg/mL (10 mg per 10 mL of solution), and for group 2, it was 5 mg/mL (10 mg per 2 mL of solution). Second, the corticosteroids effects on CTS are believed to be due to the anti-inflammation to lessen the intracarpal pressure for symptoms relief; therefore, ultrasound guidance is crucial (5). If more corticosteroid can remain closer to the inflamed MN, the better outcome it should be. However, the injectate may infiltrate more into other layers if a higher injection volume was used in group 1 compared with group 2. Moreover, in our clinical experience and cadaver studies, the fluid inside the carpal tunnel after hydrodissection may clear up within 30 min, making the final concentration of the corticosteroid reaching the under-hydrodissected MN even less (6). Assuming that the two groups have the same drug effect, there should be an additional hydrodissection effect in group 1, as was shown in a previous study with 3 months of therapeutic effect for symptom relief of CTS after 5 mL saline hydrodissection (7). However, instead of having a significant improvement in group 1 compared with group 2, group 2 had reduced symptoms and with more electrophysiological study improvements than group 1. Nevertheless, this result shows that even while using a less concentrated steroid fluid remained in the carpal tunnel next to the MN in the hydrodissected group, still with similar beneficial effects comparable with the steroid-only group, which had a much higher concentration of steroid remaining near the MN. This observation allows us to debate that hydrodissection has a greater contribution to therapeutic effect, even when a lesser concentration of steroid is injected next to the inflamed nerve.

We share a Supplementary Video 1 demonstrating the MN hydrodissection inside carpal tunnel from the ulnar side, where we observed that the final shape of the MN or its branches appeared rounded/oval instead of elliptical, and the distribution of the injectate halo is nearly uniform on the ulnar and radial sides (Figure 1).

This commentary significantly points out the critical endpoint of the ultrasound-guided hydrodissection of the MN used in CTS. All the tethering fibrotic or scar tissues over both above and below the MN, over both the ulnar and radial sides of the MN, should be hydrodissected, and the final hydrodissected MN should appear rounded/oval for the best results and prevention of recurrence. This letter also further draws attention to the possible contribution of ultrasound-guided hydrodissection of nerves on the treatment of entrapment neuropathy as in CTS.

## Author Contributions

All authors have contributed significantly in the idea formation, writing, literature collection, editing, reviewing, and final agreement of the manuscript.

## Conflict of Interest

The authors declare that the research was conducted in the absence of any commercial or financial relationships that could be construed as a potential conflict of interest.

## Publisher's Note

All claims expressed in this article are solely those of the authors and do not necessarily represent those of their affiliated organizations, or those of the publisher, the editors and the reviewers. Any product that may be evaluated in this article, or claim that may be made by its manufacturer, is not guaranteed or endorsed by the publisher.
